# Donor–acceptor Stenhouse adduct-grafted polycarbonate surfaces: selectivity of the reaction for secondary amine on surface

**DOI:** 10.1098/rsos.180207

**Published:** 2018-07-25

**Authors:** Sukhdeep Singh, Patrick Mai, Justyna Borowiec, Yixin Zhang, Yong Lei, Andreas Schober

**Affiliations:** 1 Institute of Chemistry and Biotechnology and IMN MacroNano, Technische Universität Ilmenau, Ilmenau, Germany; 2 B CUBE Center for Molecular Bioengineering, Technische Universität Dresden, Dresden, Germany; 3 Institute of Physics and IMN MacroNano, Technical University of Ilmenau, Ilmenau 98693, Germany

**Keywords:** donor–acceptor Stenhouse adducts, secondary amine, polycarbonate, amine indicator

## Abstract

Donor–acceptor Stenhouse adducts (DASAs) are gaining attention from organic and material chemists due to their visible light-stimulated photochromic properties. In this report, we present a facile method for grafting coloured triene on polycarbonate surface, without involving any pre-treatments like plasma activation, etc. The chemoselectivity of carbonate with a primary amine and Meldrum's activated furan (MAF) with polymer bound secondary amine has been exploited to graft photoswitchable DASA on the polymer surface. Primary, secondary and tertiary amine-functionalized polycarbonate surfaces have been prepared to evaluate the reactivity of amine with MAF.

## 1. Introduction

Apart from solid-phase organic synthesis, polymer functionalization plays a crucial role in the discovery of new bioactive surfaces for biomaterial and tissue engineering applications [1]. Various porous and non-porous materials require different chemical modification methodologies depending on the chemistry at the surface. Curran *et al*. [2] have shown the significant role of small organic functional groups on the surface of a material that can alter the fate of mesenchymal stem cells. Yu *et al*. [3] reported the effect of –NH_2_ functionalized surface on the adhesion and proliferation ability of human dental pulp stem cells. Additionally, a plethora of recent literature has witnessed the dependence of cell adhesion on surface chemistry [4]. Therefore, searching new methods for surface functionalization is a perpetually ongoing task.

In the field of modern bioanalytics, biosensors and diagnostic light-promoted tuning of the target molecules are playing an important role [5]. In traditional organic chemistry, light-induced isomerization phenomenon is mostly observed with high-energy photons in UV range [6]. However, for most of the biology-related applications visible or near-infrared light is considered as suitable because it does not encounter severe interference from biomolecules with high absorption characteristics [7]. Recent years have seen a rising interest of chemists to design visible light-induced photochromism [8] by tuning the molecular architecture of otherwise UV light-stimulated photoswitches [9].

In 2014, Read de Alaniz and co-workers [10] introduced donor–acceptor Stenhouse adducts (DASAs) as potential new candidates for visible light-induced photochromism. The same phenomenon was observed when these molecules were grafted on polymer surface [11]; however, the reversibility was hindered. In a significant contribution, Read de Alaniz *et al*. studied the colour reaction of different types of amines with activated furan in solution and proposed Meldrum's activated furan (MAF) conjugate as amine indicator [12]. However, no clear indication of selectivity of reaction towards a particular type of amine has been documented. In this paper, we report our observation for selective formation of DASA on polycarbonate (PC) bound secondary amines. It is a facile method for grafting photosensitive trienes on transparent PC surface, by making use of chemoselective reaction of a primary amine with carbonate and secondary amine with MAF.

## 2. Results and discussion

**Scheme 1.**
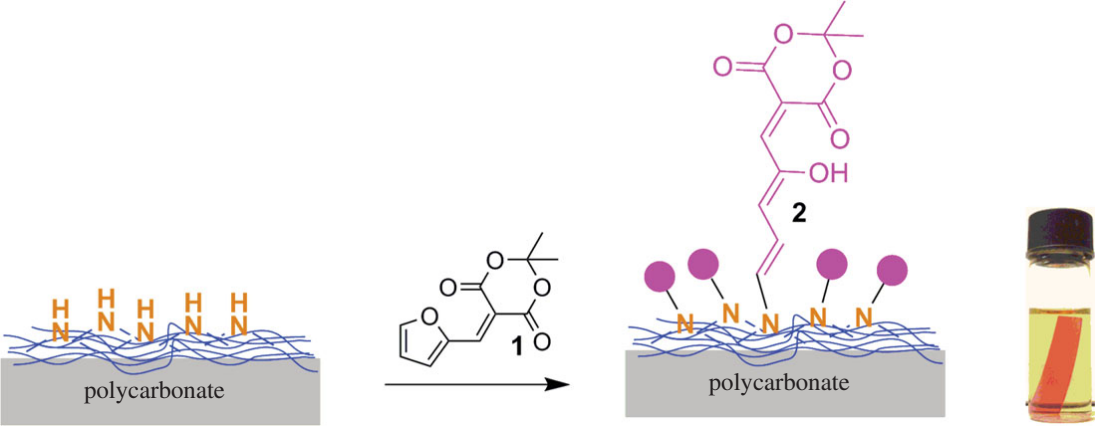
Reaction of branched polyethylene imine-functionalized surface with 5-(furan-2-ylmethylene)-2,2-dimethyl-1,3-dioxane-4,6-dione.

Among various biocompatible plastics, PC appears to be a suitable material of choice for biotechnological applications due to its (i) availability as porous membranes with controllable pore size [13], (ii) micro thermoformability that is useful for generating three-dimensional microgeometries and vessel-like structures [14] and (iii) facile surface chemistry [15]. Recently, we have reported a photoswitching behaviour of photosensitive DASA on the polymer surface. The grafting of triene on PC surface was assisted through a sandwich layer of branched polyethylene imine. The amino functionalized surface has shown a rapid reaction towards 5-(furan-2-ylmethylene)-2,2-dimethyl-1,3-dioxane-4,6-dione (MAF **1**) (scheme 1) [11].

However, due to the mixed primary, secondary and tertiary amine nature of the branched polyethylene imine, it was not clear which amine typically participated in the DASA formation. Therefore, in order to understand the reaction behaviour, we decided to investigate the interaction of particular amine-functionalized surface with MAF **1**.

**Scheme 2.**
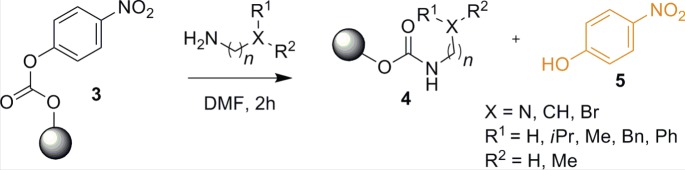
Synthesis of amine-functionalized resin beads.

In order to establish the synthesis of dedicated amine-functionalized PC surface, we have chosen polymer-bound *p*-nitrophenyl carbonate **3** as a model substance. There are two reasons for this choice. First, is the facile release of *p*-nitrophenol **5** upon nucleophilic addition of amine. Second, *in situ* appearance of yellow coloration due to release of *p*-nitrophenol under basic conditions makes this reaction a self-indicator. Therefore, monitoring reaction completion was fairly easy. Variety of commercially available diamines were reacted with resin beads in dimethylformamide (DMF) for obtaining primary, secondary and tertiary amines on the surface (scheme 2; for detail procedure, see the electronic supplementary material). In order to generate hydrophobic surface, a long aliphatic carbon chain with terminal amines was used. The amines directly attached to the aromatic ring, like aniline, have shown limited reactivity.

**Scheme 3.**
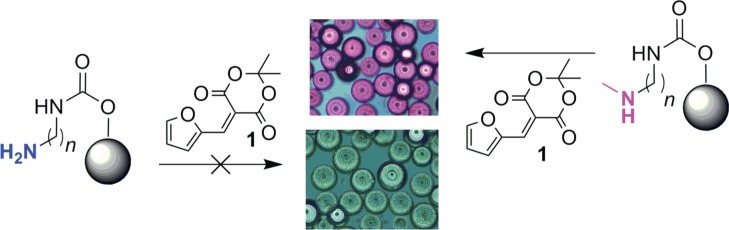
Selective coloration of secondary amine-functionalized polymer beads upon reaction with MAF.

Using the above-mentioned procedure various linker and terminal functional groups were created on the polystyrene beads **4**. In order to test the possibility of coloured DASA formation on these different types of grafted amines, they were reacted with 0.1% ethanolic solution of MAF (**1**) at room temperature. Initially, we noticed the appearance of purple coloration of all amine samples which corresponds to the formation of DASAs. Notable exceptions where when tertiary amines or amines functionalized with bromo, phenyl or aliphatic chain were used. However, a careful observation of coloured samples revealed a distinction between primary and secondary amines. In the case of secondary amine-grafted beads, the intense purple colour was developed on the surface of beads. On the other hand, primary amine-grafted polymers have shown the colour in solution and not on the surface. This could be due to self-releasing of DASA possibly by a second nucleophilic addition of amine on carbonate (see the electronic supplementary material, figure S4). While washing the reaction vessel of primary amine with water, we noticed a rapid quenching of the coloured solution. Owing to this observation, we have considered to re-examine the reaction in the presence of water. Ultimately, we observed that the treatment of primary amine-functionalized polymer beads with 0.1% MAF solution in 1 : 1 ethanol and water (at room temperature) gives no coloration. While the treatment of secondary amine surface with the same solution develops intense purple/pink coloration on polymer beads (scheme 3).

From the above-mentioned experiments, it is clear that polymer bound secondary amines via urethane linkage are showing preference for DASA formation. Our observation together with the previous investigations from Read de Alaniz *et al*. supports the use of MAF as amine indicator, specifically for secondary amines. Selective formation of triene derivative **2** (scheme 1) with a particular type of amine along with its inherent intense colour could make this type of chemistry suitable for identification of secondary amines on polymer surface. The quick reaction time and naked eye colorimetric observation make it suitable for routine analysis in surface modification organic chemistry.

**Scheme 4.**
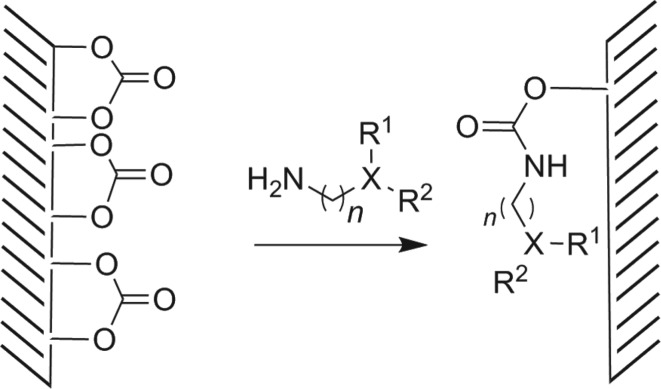
Surface modification of polycarbonate through amine–carbonate chemistry.

Owing to our ongoing research on bioactive surfaces, our main aim is to realize such selective chemistry on thermoplastic polymers like PC. This was also one of the reasons to establish the chemistry on carbonate functionalized resin beads. The major challenge in executing similar chemistry on PC surface was its fragility in organic solvents. After screening the stability of PC foil in various solutions hexane, EtOH, MeOH and *i*PrOH were selected as solvents. Similar to the resin bead chemistry (scheme 2), we used the reactivity of surface carbonate with diamines by exploring the nucleophilic addition of aliphatic amines on the carbonate linkage of the PC [15]. Therefore, in order to prepare a variety of PC surfaces, we can use differently functionalized aliphatic diamines, with an aliphatic primary amine on one end (scheme 4).

The reactivity of the amines with PC surface depends upon the nucleophilicity of respective amine; therefore, it is not possible to generalize a single reaction procedure. A concentration- and temperature-controlled functionalization of PC surface with amine in ethanol could provide crack-free smooth surfaces (table 1). In order to prepare the primary amine-functionalized surface, we treated a 1 cm^2^ PC chip with different concentrations of ethanolic solution of terminal diamines at 80°C ( table 1). Various derivatives were prepared by altering the chain length from 2 to 10 carbons (table 1, entries 1–9) between terminal amines. Increasing both temperature and concentrations adversely influences the quality of the sample surface. Similarly, other aliphatic amines like amylamine and dodecylamine yield alkane functionalized surface by treatment with 2.0% and 4.0% ethanolic solutions at 80°C, respectively (table 1, entries 14 and 15).

**Table 1.** Reaction conditions for functionalization of PC surface with different amines^a^.

entry	R^1^	R^2^	X	*n*	conc. (%)	temp. (°C)
1	H	H	N	2	1.0	80
2	H	H	N	3	1.0	80
3	H	H	N	4	1.0	80
4	H	H	N	5	2.0	80
5	H	H	N	6	2.0	80
6	H	H	N	7	2.0	80
7	H	H	N	8	2.0	80
8	H	H	N	9	3.0	80
9	H	H	N	10	3.0	80
10	*i*-Pr	H	N	2	3.0	rt
11	Me	H	N	2	1.0	rt
12	Bn	H	N	2	2.0	rt
13	Me	H	N	3	1.5	rt
14	H	H	CH	4	2.0	80
15	H	H	CH	11	4.0	80
16	Me	Me	N	2	2.0	80
17	—	—	Br	2	2.0	80
18	Ph	—	—	1	3.0	40

^a^Given conditions are optimized for preparing crack-free transparent sample.

For preparing secondary amine-functionalized surface, the samples were treated with the solutions of asymmetric diamines, where one terminal was a primary amine and the other terminal was secondary amine. Coupling of primary amine end with carbonate ultimately furnishes secondary amine-functionalized surface (table 1, entries 10–13). Compared to previously mentioned terminal diamines, these asymmetric diamines damage the surface of PC at 80°C; the best results were obtained at room temperature. As reported previously, a mixed primary, secondary and tertiary amine-functionalized surface can be prepared by treating polymer sample with 2% ethanolic solution of branched polyethylene imine at 70°C [11]. Tertiary amine-functionalized PC surface has been prepared by treating PC with 2.0% solution of dimethyl ethylene diamine at 80°C (table 1, entry 16). Additionally, bromo and phenyl functionalized surfaces were prepared by reacting PC with 2-bromoethyl hydrobromide (2%, neutralized with Et_3_N) and benzylamine (3%) at 80^o^C and 40^o^C, respectively (table 1, entries 17–18).

After preparing different derivatives of functionalized PC surfaces, the next step was to investigate the reactivity of these surfaces with MAF **1** (scheme 3). In the case of ring opening DASA formation, a colour development is expected on the surface. In order to distinguish the difference of reactivity of various functional groups, we divided the samples in three categories. Primary amine-functionalized surfaces are named as category 1 (table 1, entries 1–9), secondary amine-functionalized surfaces are category 2 (table 1, entries 10–13) and surfaces other than amine are category 3 (table 1, entries 14–18, PC, polystyrene, polypropylene and Teflon).

**Figure 1.**
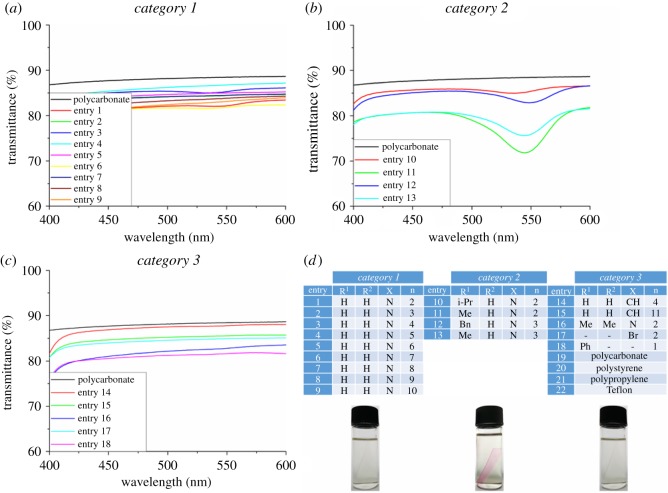
UV–visible absorption spectra of various functionalized surfaces of polycarbonate: (*a*) *category 1* are primary amine-functionalized surfaces; (*b*) *category 2* are secondary amine-functionalized surfaces; (*c*) *category 3* are surfaces with functionalization other than primary and secondary amines.

All samples were treated with 0.1% ethanol : water solution of MAF **1** at room temperature for 10 min. In order to avoid false positive, after 10 min the reagent solution was quenched with an excess of 4% ethanoic solution of polyethylene imine. In the case of category 1 where the surface has primary amines no colour appearance was observed (figure 1*d*). On the other hand, category 2 samples, with grafted secondary amine, showed development of purple colour on the surface within 10 min of the treatment with solution of MAF **1** (figure 1*d*). Benzylamine- and methylamine-functionalized surface (table 1, entries 11–13) showed rapid appearance of colour as compared to *i*-propylamine-functionalized surface (table 1, entry 10). The treatment of the category 3 samples with same solution did not develop any colour. For monitoring the changes in physico-chemical properties of the samples after treatment with **1**, we investigated the UV–visible transmission spectra (figure 1*a–c*). All the samples of three categories were compared with native PC as blank. A distinct absorption at around 540 nm was observed in the case of secondary amine-functionalized surface (figure 1*c*), which is the characteristic feature of DASA formation [11].

**Scheme 5.**
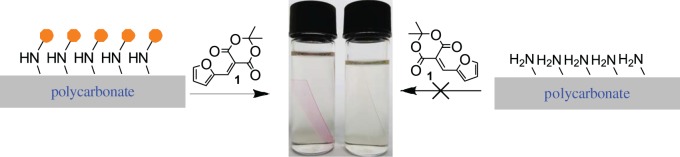
Selective colour reaction of secondary amine surface with 5-(furan-2-ylmethylene)-2,2-dimethyl-1,3-dioxane-4,6-dione.

These experiments indicate that secondary amine-grafted surfaces can be selectively functionalized with DASA triene (scheme 5), which gives strong absorption at 540 nm. The primary amine and other functionalized surfaces (category 1 and category 3) do not give an immediate colour reaction and hence DASA formation. Therefore, this method proves to be useful for selectively grafting of DASA on the polymer surfaces.

## 3. Conclusion

In the field of high-throughput discovery of bioactive surfaces, the functional group specific reactions are of high importance. The reaction of activated furans with secondary amines appears to be selective in nature. Additionally, the appearance of intense coloration due to triene formation is very useful, as it can bypass the complicated and time demanding analytics required for identification of reaction completion. We have identified the selectivity of this reaction at least on the urethane linked surface amines. Further exploration of other types of polymers, novel activated furans and effect of different linkers on this chemistry is of high interest in the field of stimuli-responsive polymers. The method mentioned in this publication is sensitive, rapid, repeatable, operationally simple and reliable. The easy preparation of MAF makes this reagent of more practical utility in common chemical laboratories. Additionally, we present an easy protocol for functionalization of PC surface with various types of amines.

## Supplementary Material

SukhdeepSingh_procedures and figures_ESM.doc

## Data accessibility

Detail of material used and analytical measurement can be found in the electronic supplementary material. No additional datasets, code and other digital research material are associated with this research.
