# Supplementary material for: Donor–acceptor Stenhouse adduct-grafted polycarbonate surfaces: selectivity of the reaction for secondary amine on surface
Source: R Soc Open Sci. 2018 Jul 25;5(7):180207. doi: 10.1098/rsos.180207 (PMC6083721; doi:10.1098/rsos.180207)
Supplement: SukhdeepSingh_procedures and figures_ESM.doc [file rsos180207supp1.doc]

## Electronic supplementary material

**Donor–Acceptor Stenhouse Adducts grafted polycarbonate surfaces: Selectivity of the reaction for secondary amine on surface**

Sukhdeep Singh^1^*, Patrick Mai^1^, Justyna Borowiec^1^, Yixin Zhang^2^, Yong Lei^3^, Andreas Schober^1^

*^1^* *Institute of Chemistry and Biotechnology & IMN MacroNano Technische Universität Ilmenau, Ilmenau, Germany*

*^2^B CUBE Center for Molecular Bioengineering, Technische Universität Dresden, Dresden, Germany*

*^3^Institute of Physics & IMN MacroNano Technical University of Ilmenau, Ilmenau 98693, Germany*

*[*sukhdeep.singh@tu-ilmenau.de*](mailto:sukhdeep.singh@tu-ilmenau.de)

**Experimental Section**

1. **Materials and methods**

Thin layer chromatography was performed on Merck TLC plates (60F254, 0.2 mm) using an appropriate solvent system. The chromatograms were visualized under UV light. All solvents and liquid reagents were dried with appropriate reagents before use. Commercially available 2-furfural, Maldrum’s acid, various types of amines, polyethyleneimine (branched), polystyrene resin beads were purchased from Sigma Aldrich. Laboratory grade solvents like ethanol, tetrahydrofuran (THF), dichloromethane (DCM) and material polycarbonate were used without any pre-treatment.

**Preparation of 5-(furan-2-ylmethylene)-2,2-dimethyl-1,3-dioxane-4,6-dione (1)**

2,2-dimethyl-1,3-dioxane-4,6-dione (1.51 g, 10.5 mmol) and 2-furaldehyde (961 mg, 10 mmol) were mixed in 30 mL H_2_O. The suspension was stirred at 70 °C for 2 h. After completion of the reaction (TLC) the precipitated solid was filtered. The collected solid was dissolved in dichloromethane and washed with 30 mL saturated aqueous NaHSO_3_, 30 mL H_2_O, 30 mL saturated aqueous NaHCO_3_, 30 mL brine. The organic layer was dried over NaSO_4_, filtered and evaporation to obtain 1.67 g of 5-(furan-2-ylmethylene)-2,2-dimethyl-1,3-dioxane-4,6-dione as a bright yellow powder. The spectroscopic data has shown good comparison with literature values^1^.


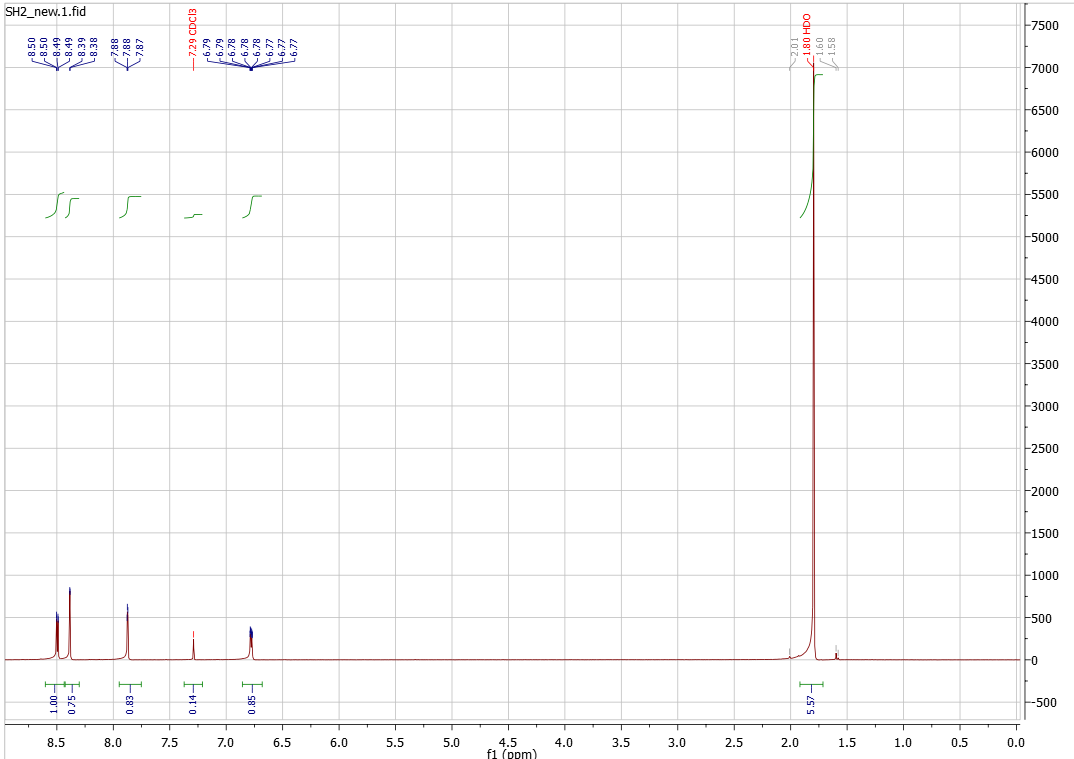


**Figure S1:** ^1^H NMR spectrum of of 5-(furan-2-ylmethylene)-2,2-dimethyl-1,3-dioxane-4,6-dione **1.**

**Reaction of secondary amine with 5-(furan-2-ylmethylene)-2,2-dimethyl-1,3-dioxane-4,6-dione; formation of DASA / 5-((2Z,4E)-5-(diethylamino)-2-hydroxypenta-2,4-dien-1-ylidene)-2,2-dimethyl-1,3-dioxane-4,6-dione**

1 mmolar solution of 5-(furan-2-ylmethylene)-2,2-dimethyl-1,3-dioxane-4,6-dione (in tetrahydrofuran (THF)) was mixed with 1 mmolar solution of diethylamine (THF). An immediate intense coloration was observed. The reaction mixture was stirred for 10 minutes and the final product was isolated by cooling. The analytical data was comparieed with literature values^1^.


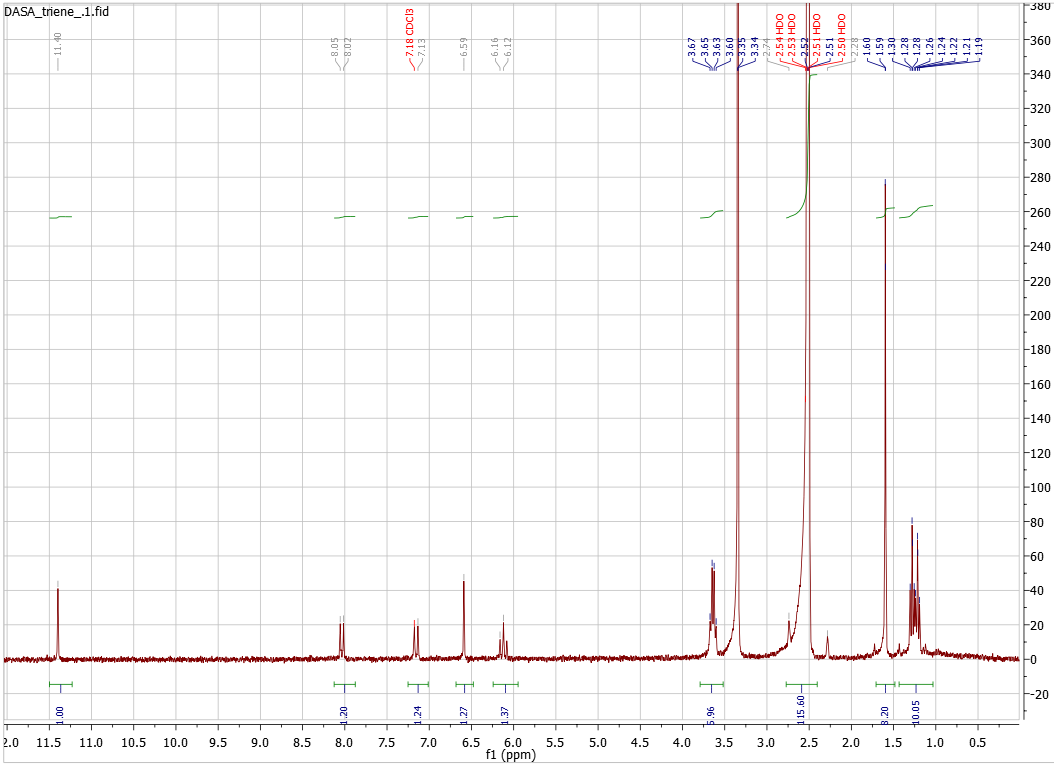


**Figure S2:** ^1^H NMR spectrum of DASA synthesized by reaction of **1** with diethyl amine.

**Amine-carbonate chemistry on polycarbonate surface**

Based on previously reported method for reactions of amine with polycarbonate^2^.we optimized surface functionalization of polycarbonate chips with minimum possible damage to the polymers. Different concentrations of amines were prepared by dissolving respective substance in 1 ml EtOH (details, manuscript table 1). A polycarbonate chip of size 1 x 1x 0.02 cm was dropped in the solution followed by heating. The vigorous shaking and heating was continued for a period of 2 hours. After cooling the reaction to room temperature the functionalized chips were washed 3 times with EtOH (4 mL) under ultrasonication and dried with nitrogen gas. The surface functionalization has been analysed with contact angle measurements and by a dying test described below (figure S3).

**Procedure of colour development on secondary amine functionalized surface.**

A secondary amine functionalized polycarbonate sample were suspended in 1 mL 0.1% ethanol/water (1:1) solution of 5-(furan-2-ylmethylene)-2,2-dimethyl-1,3-dioxane-4,6-dione. The solution was vigorously shacked at room temperature for 10 minutes. The reaction performance was confirmed by appearance of pink colour on the polymer chips (as shown in figure above). The functionalized chips were washed by three cycles of ultrasonication in EtOH (4 mL) and subsequently dried with nitrogen gas. The surface functionalization has been confirmed by contact angle measurements and colour development on the chip.

**Procedures for grafting diamine on the polymer-bound p-nitrophenyl carbonate beads:**

20 mg of p-nitrophenyl carbonate bound polystyrene beads were suspended in 1 mL solution of dimethylformamide (DMF). The polymer beads were soaked in this solution for 15 minutes to enhance the swelling. After this time, desired amine (50 μL) was added to the solution and the reaction was shacked at room temperature for 2 h. Polymers beads were filtered and washed with ethanol to remove unreacted amine and *p*-nitrophenol. The presence of amine on the polymer surface was analysed with dying method, as described in analysis section (figure S3).

1. **Analysis**

^1^H/^13^C NMR (300/75 MHz; CDCl_3_, DMSO, MeOD-d^4^) spectra were recorded using commercial available deuterated solvents on a multinuclear spectrometer Bruker 300 MHz. Data analysis was performed using the MestReNova v8.1.0-11315 software. IR spectra were recorded using a Jasco FTIR 6300 spectrophotometer. An Aligant 1200 series HPLC system coupled with an esquire 6000 mass spectrometer was used to investigate the reactions in LCMS. To measure contact angles we used a syringe to deposit a 8 µl droplet of water on the surface of PC and a camera to record the shape of the droplet and measure the contact angle based on a drop shape analysis (DAS 10 MK2 Krüss).

**Procedure for detection of amines on the polymer surface**: 20 mg of amine functionalized polymer beads were incubated with 0.1% ethanolic solution of dansyl chloride for 2 hours at room temperature. After this time, the unreacted dansyl chloride solution was removed and the polymer was washed with ethanol 4-5 times. In order to observe the functionalization, the samples were exposed with 365nm UV torch. The amine functionalized surfaces were showing significant fluorescence. As a control native polymer was also treated with same procedure, but no fluorescence was observed.

**
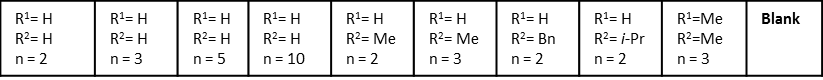
**

**
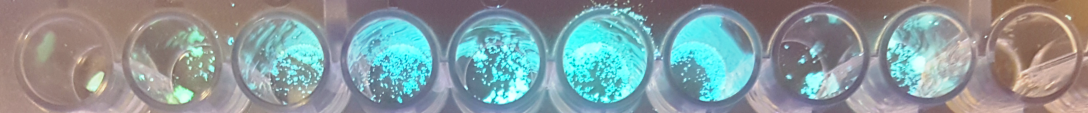
**

**Figure S3:** labelling of amine functionalized surfaces with dansyl fluorophore confirming the presence of amine groups on the surface.

**Table S1:** Comparison of the contact angle measured after the functionalization steps with blank PC (Water contact angle 88.8^o^).

| Amine functionalized polycarbonate surface | Water contact angle (^o^) | DASA functionalized polycarbonate surface | Water contact angle (^o^) |
| --- | --- | --- | --- |
|  | 73.1 |  | 80.5 |
|  | 76.6 |  | 55.9 |
|  | 73.7 |  | 61.0 |
|  | 61.0 |  | 63.3 |
|  | 60.2 |  | 75.1 |
|  | 64.7 |  | 67.7 |

1. **Hypothesis**

According to our observations, where primary amine functionalized beads gave color in the solution and secondary amine functionalized beads on the surface, we believe that it is due to high nucleophilicity of neighboring amine for carbamoyl linkage. In case of secondary amine functionalized surface it generates tertiary amine, which has no further reactivity to the carbamoyl group (figure S4).

**
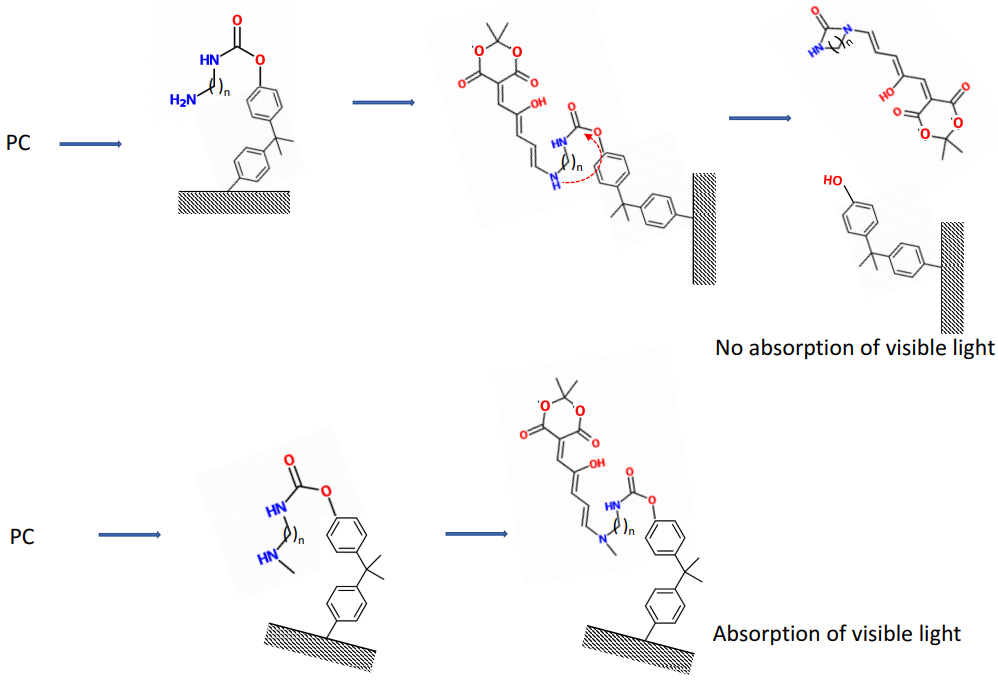
**

**Figure S4**: explanation of the colour development on the surface of secondary amine *vs* primary amine.

In a second hypothesis for the selectivity of the secondary amine to the activated furan, we assume that it might be due to relative softness (more electron density) of the secondary amines over primary amine. Due to +I effect of aliphatic amines the secondary amine could be consider as soft nucleophile as compare to primary amine. On the other hand, due to extended π electrons cloud over furan ring, which is easy to distort, it could be considered soft electrophile. Hence, we assume that due to soft-soft interactions of relatively polarizable electron cloud on both chemical species, new bond is formed.

1. **References**

1. S. Helmy, S. Oh, F. A. Leibfarth, C. J. Hawker and J. Read de Alaniz, *The Journal of Organic Chemistry*, 2014, **79**, 11316-11329.

2. C. J. R. and J. W. J., *Journal of Polymer Science Part C: Polymer Symposia*, 1968, **24**, 15-23.
